# Plasma lipid profiles in early adulthood are associated with epigenetic aging in the Coronary Artery Risk Development in Young Adults (CARDIA) Study

**DOI:** 10.1186/s13148-021-01222-2

**Published:** 2022-01-31

**Authors:** Tao Gao, John T. Wilkins, Yinan Zheng, Brian T. Joyce, David R. Jacobs, Pamela J. Schreiner, Steve Horvath, Philip Greenland, Donald Lloyd-Jones, Lifang Hou

**Affiliations:** 1 Department of Preventive Medicine, Northwestern University Feinberg School of Medicine, 680 N. Lake Shore Drive, Suite 1400, Chicago, IL 60611 USA; 2 Center for Global Oncology, Institute for Global Health, Northwestern University Feinberg School of Medicine, Chicago, IL USA; 3 Department of Medicine (Cardiology), Northwestern University Feinberg School of Medicine, Chicago, IL USA; 4 Division of Epidemiology and Community Health, University of Minnesota School of Public Health, Minneapolis, MN USA; 5 Department of Human Genetics, David Geffen School of Medicine, University of California Los Angeles, Los Angeles, CA USA; 6 Department of Biostatistics, Fielding School of Public Health, University of California Los Angeles, Los Angeles, CA USA

**Keywords:** Lipid, GrimAge acceleration, Coronary artery calcification

## Abstract

**Background:**

GrimAge acceleration (GAA), an epigenetic marker that represents physiologic aging, is associated with atherosclerotic cardiovascular disease. However, the associations between early adulthood lipid levels and GAA in midlife are unknown. Also, it is unknown whether GAA mediates the associations between lipid levels in young adults and subclinical atherosclerosis in midlife.

**Results:**

We estimated measures of epigenetic age acceleration in 1118 White and Black participants from the Coronary Artery Risk Development in Young Adults (CARDIA) Study at examination years (Y) 15 and 20. We used multivariable regression models to examine associations of Y15 and Y20 GAA estimates with plasma lipid levels measured at prior examination years (Y0, Y5, and Y10) and concurrently: triglycerides (TG), low-density lipoprotein cholesterol (LDL-C), and high-density lipoprotein cholesterol (HDL-C) levels. Mediation analysis was used to assess the extent to which GAA may mediate associations between plasma lipids and coronary artery calcification (CAC). In our study each 1-SD higher cumulative TG level was associated with an average 0.73 ± 0.12 years older GAA. Each 1-SD higher cumulative HDL-C level was associated with an average 0.57 ± 0.17 years younger GAA. Stratified analyses showed that the associations between TG and GAA were stronger among female and Black participants and the associations between HDL-C and GAA were stronger among female and White participants. GAA statistically mediated 17.4% of the association of cumulative TG with CAC.

**Conclusions:**

High TG and low HDL-C in early adulthood are associated with accelerated epigenetic aging by midlife. Increased epigenetic age acceleration may partially mediate the associations between high TG levels and the presence of subclinical atherosclerosis.

**Supplementary Information:**

The online version contains supplementary material available at 10.1186/s13148-021-01222-2.

## Background

Atherosclerotic cardiovascular disease (ASCVD) is the leading cause of death in the USA and worldwide, and a major cause of disability [1]. The cumulative exposure to atherogenic lipoproteins with aging is a primary cause of atherosclerosis and subsequent ASCVD events [2, 3]. The presence or absence of other traditional risk factors (hypertension, diabetes, tobacco use) explains some of the inter-individual differences in observed risk, but heretofore incompletely understood molecular characteristics may also explain some of the difference in lipid-associated risks observed across individuals as well.

Epigenetic modifications like DNA methylation (DNAm) patterns are influenced by genetic and environmental factors and have been used for predicting chronological age [4, 5]. Several epigenetic biomarkers using weighted averages of methylation levels at specific CpG sites have been proposed to measure “epigenetic age,” and various studies have shown that these measures are prospectively associated with age-related diseases like cancer, type 2 diabetes, cardiovascular diseases, and all-cause mortality [4–8]. Studies from our own group also showed that epigenetic age measures were associated with metabolic syndrome severity score [9]. In these studies, various versions of epigenetic age indexes were used including intrinsic and extrinsic epigenetic age acceleration (IEAA and EEAA) which were designed to represent biological and immune system aging, respectively. One of newly developed indexes of epigenetic age, GrimAge, predicts lifespan as well as duration of healthy longevity [10]. Statistical regression has also been used to calculate the residual of observed vs. predicted GrimAge to yield GrimAge acceleration (GAA), an index of greater/lower than expected epigenetic age for a given chronological age [10]. The cumulative exposure to environmental factors that mediate lipid levels as well as lipid levels themselves may lead to epigenetic modifications that accelerate GrimAge [11]. Studies have shown that blood triglycerides (TG) and HDL cholesterol (HDL-C) are both significantly associated with old indexes of epigenetic age measures like extrinsic epigenetic age acceleration [12, 13]. Given the strong associations between lipid levels, aging, and ASCVD we hypothesized that measures of GrimAge in the middle life would be associated with lipid levels earlier in life, and that GAA may mediate the associations between lipid levels and subclinical atherosclerosis.

With longitudinal measurements of lipid profiles during young adulthood and repeated measurements of DNA methylation and subclinical atherosclerosis measures in midlife, the Coronary Artery Risk Development in Young Adults (CARDIA) Study provides a unique opportunity to study the associations between lipids in early adulthood, epigenetic age measurements, and subclinical atherosclerosis in midlife [14]. Thus, we aimed to quantify the associations between plasma lipid profiles and GAA and the mediating effects of GAA on the association between plasma lipid profiles and midlife subclinical cardiovascular disease-coronary artery calcification (CAC).

## Results

Blood methylation measurements were available for 1118 CARDIA participants. The study sample was 51% females, 41% Black, and the average age was 41 ± 4 years at the Y15 visit. Over sixty percent of the study participants were never-smokers. Table 1 shows descriptive analyses of participant characteristics at the Y15 and Y20 visits.

**Table 1:** Study participant characteristics by study year^a^

Characteristics	Y15	Y20
*N*	1042	957
Age, mean (SD), y	40.9 (3.5)	45.4 (3.5)
Center, *n* (%)		
Birmingham, AL	255 (24.5)	222 (23.2)
Chicago, IL	225 (21.6)	208 (21.7)
Minneapolis, MN	278 (26.7)	258 (27.0)
Oakland, CA	284 (27.3)	269 (28.1)
Sex, *n* (%)		
Male	507 (48.7)	467 (48.8)
Female	535 (51.3)	490 (51.2)
Race, *n* (%)		
White	618 (59.3)	564 (58.9)
Black	424 (40.7)	393 (41.1)
Education, mean (SD), y	15.1 (2.5)	15.0 (2.5)
Smoking status, *n* (%)		
Never	653 (62.8)	570 (60.2)
Former	181 (17.4)	191 (20.2)
Current	206 (19.8)	186 (19.6)
Alcohol, mean (SD), mL/day	12.1 (22.6)	11.1 (18.4)
Physical activity, mean (SD), total intensity score	350.0 (274.7)	348.6 (276.1)
Triglycerides, mean (SD), mg/dL	110.9 (93.4)	119.0 (95.4)
LDL cholesterol, mean (SD), mg/dL	114.3 (31.6)	112.2 (32.7)
HDL cholesterol, mean (SD), mg/dL	50.1 (14.1)	53.2 (16.7)
Cumulative triglycerides, mean (SD), mg/dL * year	1394.9 (1008.9)	2007.9 (1398.9)
Cumulative LDL cholesterol, mean (SD), mg/dL * year	1674.0 (404.7)	2252.5 (529.7)
Cumulative HDL cholesterol, mean (SD), mg/dL * year	770.0 (184.0)	1026.0 (251.7)
Grim AA, year	− 0.05 (4.58)	− 0.09 (4.63)
CAC, prevalence, *n* (%)	92 (10.0)	178 (20.0)

^a^Among the 1118 participants who had either Y15 or Y20 DNA methylation, 881 participants had DNA methylation at both Y15 and Y20

**Fig. 1:**
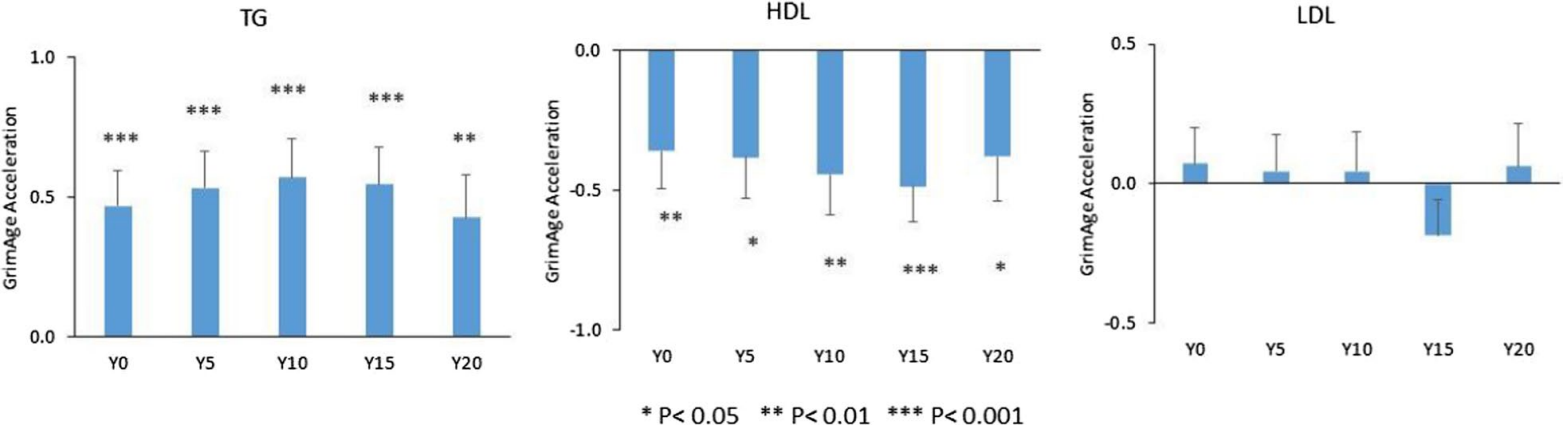
Associations between lipid profiles and GrimAge acceleration by study year. Difference in GrimAge acceleration (in years) per each 1-SD higher lipid levels for all participants. Models adjusted for center, sex, race, education, alcohol drinking, and physical activity

**Fig. 2:**
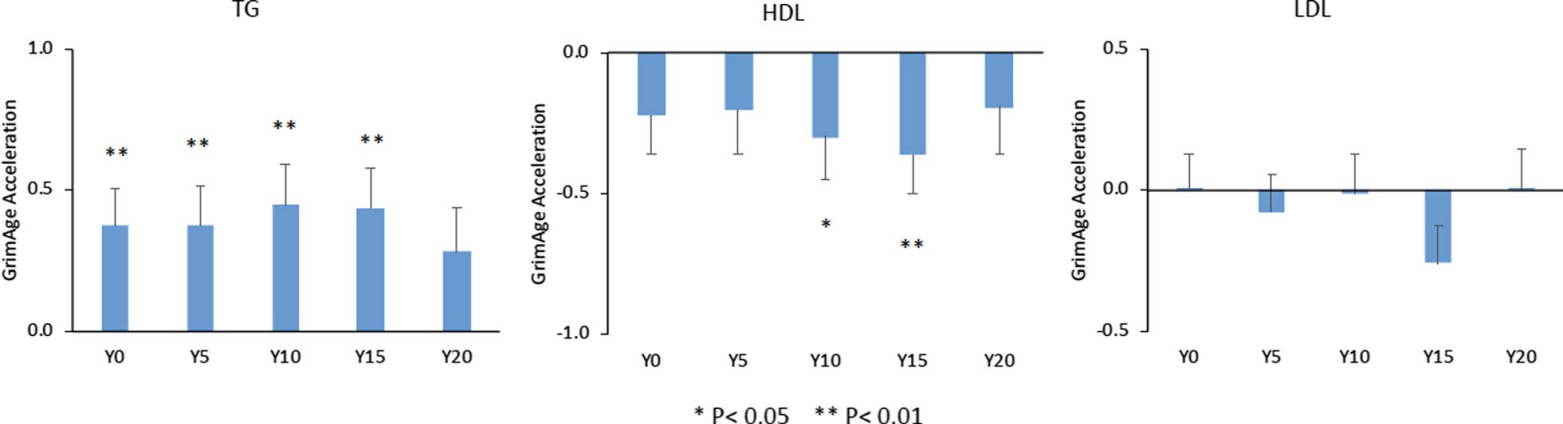
Associations between lipid profiles and GrimAge acceleration by study year further adjusted for BMI. Difference in GrimAge acceleration (in years) per each 1-SD higher lipid levels for all participants. Models adjusted for center, sex, race, education, alcohol drinking, physical activity, and BMI

Mean plasma TG and LDL-C levels increased, while HDL-C levels decreased over the 20y of CARDIA follow-up (Additional file 1: Fig. S1). TG levels at each CARDIA visit were positively associated with GAA. Each 1-SD higher TG level was associated with older GAA for an average 0.47 ± 0.13 years at Y0, 0.53 ± 0.13 years at Y5, 0.57 ± 0.14 years at Y10, 0.55 ± 0.13 years at Y15, and 0.43 ± 0.15 years at Y20, separately. Plasma HDL-C levels at each CARDIA visit were inversely associated with GAA. Each 1-SD higher HDL-C level was associated with younger GAA for an average 0.36 ± 0.13 years at Y0, 0.38 ± 0.15 years at Y5, 0.45 ± 0.14 years at Y10, 0.49 ± 0.13 years at Y15, and 0.38 ± 0.16 years at Y20, separately. There were no significant associations between plasma LDL-C level and GAA regardless of study year (Fig. 1). As a sensitivity analysis, the associations of TG, LDL-C, and HDL-C with GAA were further adjusted for BMI. After adjusting for BMI, the associations of TG and HDL-C with GAA were partially attenuated. Each 1-SD higher TG level was associated with older GAA ranging between 0.38 and 0.45 years for Y0 to Y15. For HDL-C, each 1-SD higher HDL-C level was associated with younger GAA of 0.30 and 0.36 years for Y10 and Y15 separately (Fig. 2).

**Fig. 3:**
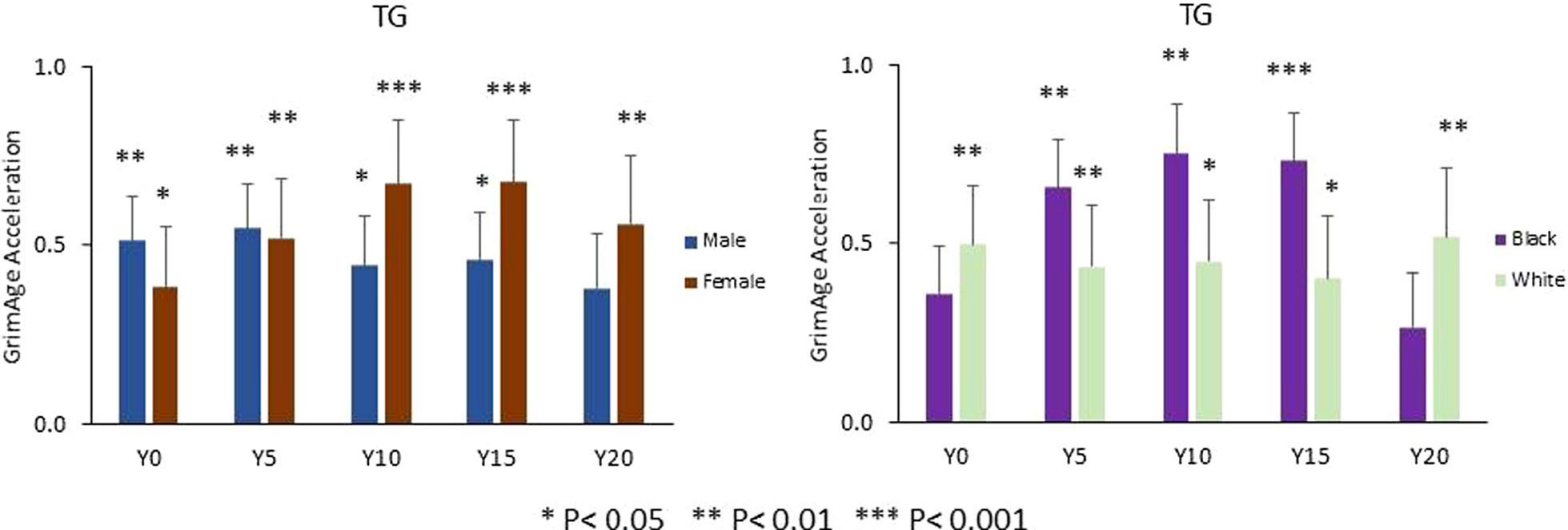
Sex- and race-specific associations between TG levels and GrimAge acceleration by study year. Difference in GrimAge acceleration (in years) per each 1-SD (within-strata) higher TG. Models adjusted for center, education, alcohol drinking, and physical activity

When stratified by sex, TG levels were more strongly associated with GAA in females than in males. For females, each 1-SD higher TG level was associated with older GAA for an average 0.38 ± 0.17 years at Y0, 0.52 ± 0.17 years at Y5, 0.68 ± 0.18 years at Y10, 0.68 ± 0.17 years at Y15, and 0.56 ± 0.20 years at Y20, separately. For males, each 1-SD higher TG level was associated with an average of 0.38 to 0.54 years older GAA between Y0 and Y20. When stratified by race, TG levels in Black participants were more strongly associated with GAA than in White participants. For Black participants, each 1-SD higher TG level was associated with older GAA for an average 0.36 ± 0.21 years at Y0, 0.66 ± 0.21 years at Y5, 0.75 ± 0.23 years at Y10, 0.73 ± 0.20 years at Y15, and 0.26 ± 0.26 years at Y20, separately. For White participants, each 1-SD higher TG level was associated with an average of 0.40 to 0.51 years older GAA between Y0 and Y20 (Fig. 3).

**Fig. 4:**
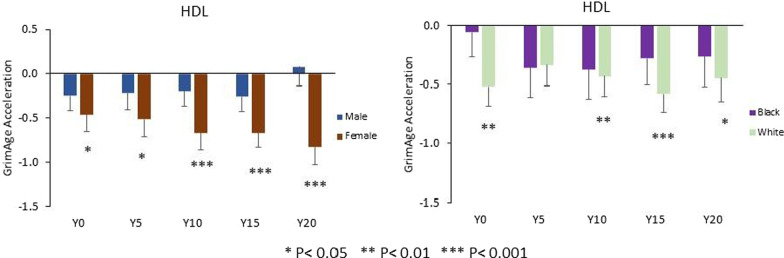
Sex- and race-specific associations between HDL-C levels and GrimAge acceleration by study year. Difference in GrimAge acceleration (in years) per each 1-SD higher HDL-C (within-strata SD). Models adjusted for center, education, alcohol drinking, and physical activity

When stratified by sex, HDL-C was also more strongly associated with GAA in females than in males. In females, each 1-SD higher HDL-C level was associated with younger GAA for an average 0.46 ± 0.20 years at Y0, 0.51 ± 0.20 years at Y5, 0.67 ± 0.20 years at Y10, 0.66 ± 0.17 years at Y15, and 0.83 ± 0.20 at Y20, separately. In males, each 1-SD higher HDL-C level was associated with an average − 0.08 to 0.25 years younger GAA between Y0 and Y20. When stratified by race, HDL-C in White participants was more strongly associated with GAA than in Black participants. In White participants, each 1-SD higher HDL-C level was associated with younger GAA for an average 0.52 ± 0.17 years at Y0, 0.34 ± 0.18 years at Y5, 0.44 ± 0.17 years at Y10, 0.58 ± 0.16 years at Y15, and 0.45 ± 0.20 years at Y20, separately. In Black participants, each 1-SD higher HDL-C level was associated with an average 0.06 to 0.37 years younger GAA between Y0 and Y20 (Fig. 4).

Cumulative TG from Y0-15 was strongly associated with GAA. Each 1-SD higher cumulative TG level was associated with an average 0.73 ± 0.12 years older GAA, especially for females compared to males (0.90 ± 0.19 years vs. 0.61 ± 0.21 years) and Black compared to White participants (1.11 ± 0.21 years vs. 0.52 ± 0.19 years). Cumulative HDL-C from Y0-15 was associated with GAA in which each 1-SD higher cumulative HDL-C level was associated with an average 0.57 ± 0.17 years younger GAA, especially for females compared to males (− 0.78 ± 0.22 years vs. − 0.32 ± 0.20 years) and Black participants compared to White participants (− 0.64 ± 0.30 years vs. − 0.50 ± 0.17 years) (Table 2).

**Table 2:** Associations between cumulative TG and HDL-C from Y0 to Y15, and GrimAge acceleration^a,b^

		Estimate (yr/SD)	Standard error	95% CI		*P*
TG	Total	0.730	0.122	0.426	1.034	< 0.0001
	Male	0.614	0.205	0.205	0.956	0.0025
	Female	0.900	0.189	0.521	1.278	< 0.0001
	Black	1.110	0.211	0.687	1.585	< 0.0001
	White	0.518	0.194	0.130	0.842	0.0049
HDL-C	Total	− 0.569	0.165	− 0.881	− 0.275	0.0002
	Male	− 0.321	0.196	− 0.695	0.053	0.09
	Female	− 0.776	0.215	− 1.189	− 0.380	0.0002
	Black	− 0.643	0.297	− 1.203	− 0.066	0.03
	White	− 0.503	0.174	− 0.851	− 0.155	0.005

^a^Difference in GrimAge acceleration (in years) per each 1-SD higher cumulative TG/HDL-C

^b^Model adjusted for center, sex, race, education, alcohol drinking, and physical activity

For the association between TG and Y25 CAC, both 20-year cumulative TG and TG at each individual CARDIA visit were associated with Y25 CAC (OR: 1.37, 95% CI: 1.12–1.68 for cumulative TG). Neither cumulative HDL-C nor HDL-C at any given examination was associated with Y25 CAC (Table 3).

**Table 3:** Associations between TG and HDL-C and coronary artery calcification^a^

	OR (per SD)	95% CI		*P*
Cumulative TG	1.370	1.116	1.681	0.003
TG Year0	1.259	1.016	1.559	0.035
TG Year5	1.236	1.010	1.514	0.040
TG Year10	1.237	1.005	1.524	0.045
TG Year15	1.370	1.132	1.656	0.001
TG Year20	1.372	1.127	1.670	0.002
Cumulative HDL-C	0.830	0.647	1.065	0.143
HDL-C Year0	0.936	0.750	1.169	0.559
HDL-C Year5	0.918	0.731	1.152	0.460
HDL-C Year10	0.842	0.660	1.074	0.166
HDL-C Year15	0.781	0.600	1.018	0.068
HDL-C Year20	0.861	0.663	1.119	0.264

^a^Model adjusted for center, sex, race, education, alcohol drinking, and physical activity

In mediation analysis, GAA mediated about 10–25% of the effect of TG on CAC at Y25, depending on the year of TG measurement, and 17.4% of the effect of cumulative lipids from Y0-20. The mediation effect of GAA was higher for TG in early midlife (22.5% at Y5 and 25.2% at Y10, corresponding to mean participant ages of 30 and 35, respectively) compared with TG at Y0 (14.0%), Y15 (14.3%), and Y20 (11.2%) (Table 4).

**Table 4:** Mediation analysis of GrimAge acceleration for the TG/HDL-C and CAC associations^a^

	Total effect	Mediation effect	Direct effect	Percent mediated (%)	*P* value
TG Year0	0.023	0.003	0.019	14.0	0.070
TG Year5	0.023	0.006	0.017	22.5	0.046
TG Year10	0.024	0.006	0.017	25.2	0.041
TG Year15	0.030	0.005	0.026	14.3	0.005
TG Year20	0.031	0.004	0.027	11.2	0.013
Cumulative TG	0.030	0.006	0.024	17.4	0.005
HDL-C Year0	− 0.014	− 0.004	− 0.009	15.3	0.583
HDL-C Year5	− 0.018	− 0.007	− 0.011	23.9	0.469
HDL-C Year10	− 0.034	− 0.007	− 0.027	19.4	0.194
HDL-C Year15	− 0.048	− 0.012	− 0.036	25.3	0.117
HDL-C Year20	− 0.028	− 0.007	− 0.021	19.4	0.312
Cumulative HDL-C	− 0.037	− 0.010	− 0.027	25.1	0.181

^a^Model adjusted for center, sex, race, education, alcohol drinking, and physical activity

## Discussion

To our knowledge this study is the first to assess the prospective association between plasma lipid profiles and GAA. We found that both plasma TG and HDL-C (but not LDL-C) longitudinally measured from young adulthood to midlife (mean age 25 to 45) were associated with midlife GAA. The association between both plasma TG and HDL-C and midlife GAA was partially attenuated after adjusting for BMI. Furthermore, the associations between TG and GAA were generally consistent for both sexes and races studied. The associations of TG with GAA were stronger among women and Black participants, and the associations of HDL-C with GAA were stronger among women and White participants. Associations were generally consistent across all study years examined, with the strongest associations occurring with lipid measurements taken closer in time to DNA methylation measurement. We likewise found associations similar in magnitude with cumulative lipids, overall and in most race and sex subgroups, suggesting that GAA may partially integrate lifetime lipid burden. Further, midlife GAA mediated some of the associations between earlier TG level and later CAC, suggesting that GAA might have use as a biomarker of early life atherosclerosis risk and could become a potential target for therapy if the mechanism is elucidated.

Several studies have explored the relationships between epigenetic age acceleration measures and lipid profiles. A study by Horvath et al. of ~ 1500 older (age between 50 and 80) Women's Health Initiative (WHI) participants found significant associations between extrinsic epigenetic age acceleration and TG, but not for HDL-C [12]. Irvin et al. found that extrinsic epigenetic age acceleration was inversely associated with HDL-C in 830 White participants (mean age 50) from the Genetics Of Lipid Lowering Drugs and diet Network (GOLDN) study [13]. In our study of middle-aged participants with longitudinal lipid levels through young adulthood, we found consistent results with these prior reports. The fact that our findings were prospective, and generally consistent even earlier in the study (5–20 years before methylation measurement), suggests that GAA may capture cardiovascular health early in the life course. As such, the role for GAA in ASCVD risk prediction in younger adults should be explored, particularly if greater GAA precedes the development of currently used risk stratification tools like CAC.

In recent years, a rapidly growing body of literature suggests that the associations between TG levels and ASCVD are causal; however, the biologic mechanisms underlying these associations are complex and pathways that mediate TG metabolism have direct effects upon ASCVD risk (e.g., diabetes) [15–18]. As a surrogate of several plasma proteins related to both morbidity and mortality, GrimAge may reflect the multiple effects of TG on cardiovascular health. For HDL-C, most current literature suggests that HDL-C serves as an integrative biomarker of cardiometabolic health, but is not in of itself a causal factor for ASCVD [19, 20]. Thus, the associations between HDL-C and GAA may be mediated by the myriad of other environmental and metabolic determinants of HDL-C level. For example, excess adiposity likely explains some of the associations between lipid levels and GAA observed as BMI is positively correlated with TG and negatively correlated with HDL-C [21] and statistical adjustment for BMI partially attenuated the associations between lipid levels and GAA. Interestingly, LDL-C was not associated with midlife GAA. LDL-C has a direct causal relationship with atherosclerosis that is largely determined by the aggregate exposure to apolipoprotein B-containing lipoproteins across the life course [22, 23]. We posit the differential mediation effects observed in this study reflect the distinct biologic pathways that mediate the associations between TG, LDL-C, HDL-C, and ASCVD. GrimAA can work as an integrative marker for cardiovascular health, as it captures multiple ASCVD-related processes similarly to the two lipids measures (TG, HDL-C) associated with it. Our former study has shown that GAA are associated with AHA Life’s Simple 7 metrics, which includes four health factors (blood pressure, total cholesterol, BMI, and fasting blood glucose) and three health behaviors (smoking, physical activity, and diet) [24]. While total cholesterol may possibly contribute to the overall effect of Life’s Simple 7 metrics, our current study showed both TG and HDL-C individually affected GAA.

In our study, the associations between TG and midlife GAA were stronger in women and Black participants, while the associations between HDL-C and midlife GAA were stronger in women and White participants. TG and HDL-C levels are known to differ between race and sex groups [25]. Furthermore, race and sex differences in DNA methylation have been reported, though the reasons for these differences are not well understood [12, 26]. For example, Zhang et al. have shown that women have a significantly lower level of global methylation than men [26] and Hsiung et al. showed lower levels of global methylation in White as compared to Black participants [27]. Thus, it is possible that the differences we observed in GAA between race and sex groups are due to different environmental exposures (or other unmeasured confounders) between groups affecting TG, HDL-C, and GAA. Lifestyle or similar geography-independent exposures (e.g., dietary patterns, obesity) may also mediate TG and HDL-C levels as well as subclinical atherosclerosis development [28, 29], leading to the differential associations by sex and race along similar lines to those that we report in this study (i.e., higher intake of vegetables and fruits by females may lead to lower TG levels as well as decreased GAA) [30, 31]. Alternatively, these sex- and race-specific associations may point to differences in the underlying biology of cardiovascular disease in these subpopulations which may in turn shed light on mechanisms of race and gender disparities in cardiovascular disease. Further exploration of this hypothesis may provide additional knowledge for the early detection of cardiovascular disease in at-risk subpopulations, including methods for targeted prevention of sex and race disparities in cardiovascular outcomes.

Our mediation analysis showed that GAA mediated about 20% of the effect of plasma TG on CAC. This is consistent with the currently available literature. Blood TG has been reported to be associated with extrinsic epigenetic age acceleration [12, 13]. Lu et al. observed that GAA is significantly associated with coronary heart disease as well as congestive heart failure [10]. Further, GAA is calculated using DNAm-based surrogate markers of seven plasma proteins, and several of these markers have well-described roles in the pathogenesis of atherosclerosis [10]. For example leptin, which is secreted from adipose cells, has been shown to affect cardiovascular homeostasis and stimulate both vascular inflammation and smooth muscle hypertrophy, all of which may contribute to atherosclerosis and coronary heart disease [32, 33]. Similarly, PAI-1, which was also used to derive GAA, is strongly associated with markers of obesity and insulin resistance, and it is believed to have a role in the initiation of atherosclerosis as well as atherothrombotic events [34, 35]. As a surrogate to these plasma proteins, GAA may be a useful biomarker (or supplement) to these other factors. We also note that GAA mediated a higher proportion of the TG-CAC association when TG was measured earlier in life. This suggests that GAA may capture damage associated with long-term lipid exposure, and/or that the period represented by study years 0–10 is particularly important for later CAC development in a way that GAA captures. Thus, ASCVD long-term risk assessment using HDL-C and TG levels might be better informed by the simultaneous assessment of GAA. However, further research is needed to replicate these associations and determine the mechanistic underpinnings of these associations and the clinical test characteristics of GAA before these can be considered for clinical use.

The findings we report should be interpreted in the context of the study’s strengths and limitations. The strengths of this analysis include the high-quality phenotypic data from CARDIA obtained longitudinally from young adulthood through middle age. Furthermore, the intentional oversampling of Black participants as well as representative sampling of women in CARDIA allows for sex- and race-specific analyses. However, our study has limitations as well. First, in our study epigenetic profiling was performed using blood samples and tissue-specific methylation was not examined, which limits our ability to make inferences on clinical ASCVD. Second, given the CARDIA study design, we were unable to study racial/ethnic minority groups outside of Whites and Blacks; thus, our results may not be generalizable to other populations. Third, there are other unmeasured confounding factors which may affect the associations between lipids and GAA including diet, etc.

In conclusion, we observed that elevated TG and low HDL-C levels in young adulthood are associated with accelerated midlife epigenetic aging, and epigenetic aging mediates some of the well-described associations between elevated TG levels in early life and subclinical atherosclerosis in middle age. These findings suggest that maintaining optimal lipid levels in early adulthood may help to slow epigenetic aging, which reflects delays in the onset of age-related diseases like atherosclerosis.

## Methods

### Study sample

The CARDIA Study is a multicenter, longitudinal, population-based cohort of 5115 Black and White men and women, who were ages 18 to 30 years at year 0 (1985 to 1986) and who were recruited from 4 urban areas (Birmingham, Alabama; Chicago, Illinois; Minneapolis, Minnesota; and Oakland, California). Within each center, the sample was designed to have approximately equal numbers of participants by sex, race (Black or White), age (18 to 24 years or 25 to 30 years), and education (high school graduate or less, or beyond high school). Eight follow-up examinations have been conducted at years 2, 5, 7, 10, 15, 20, 25, and 30 (2015 to 2016), with 71% of the surviving cohort attending the year 30 (Y30) examination [14, 36]. For the current study, 1200 participants who had available whole blood at years 15 and 20 were randomly selected for methylation profiling. All examinations were approved by the institutional review boards at each participating institution, and all participants gave written informed consent.

### Lipid measurements

Fasting blood samples were drawn at each CARDIA examination, and measurements on plasma stored at − 70 °C were taken at the Northwest Lipid Research Lab, University of Washington. Studies have shown that lipids were very stable when stored at − 70 to − 80 °C [37–39]. Total cholesterol and TG were measured enzymatically, HDL-C was determined by precipitation with dextran sulfate magnesium chloride, and LDL cholesterol was calculated using the Friedewald equation [40]. We also calculated annualized cumulative lipids by averaging the product of mean lipid levels and the time interval between 2 consecutive examinations over 15/20 years (for participants with no missing examinations during the 15/20 years of follow-up).

### Epigenetic age acceleration

DNA was extracted from whole-blood white cells, and one microgram of DNA underwent bisulfite conversion. DNA methylation was measured using Infinium Methylation EPIC BeadChip (EPIC array). GrimAge is a weighted sum of methylation levels at 1030 CpGs sites [10] that are surrogates of seven plasma proteins (including adrenomedullin, beta-2 microglobulin, cystatin C, growth differentiation factor 15, leptin, plasminogen activation inhibitor 1, and tissue inhibitor metalloproteinase 1) and smoking pack-years; GrimAge predicts time to all-cause mortality and was calculated for each participant. This DNAm-based biomarker of mortality is named "GrimAge" because high values are grim news, with regard to mortality/morbidity risk. GrimAge acceleration (GAA) was also calculated as the residuals from simple linear regression model of methylation age on chronological age (making GAA independent of chronological age).

### Measurement of CAC by CT scan

A standard protocol for non-contrast CT scan was used to measure CAC [41], and a single CT scan was performed at Y25. The Agatston score was calculated corrected for slice thickness, with a minimum calcification area of 1.87 mm^2^ and attenuation threshold of 130 or more Hounsfield units on a dedicated computer workstation (TeraRecon) [42] Robustness of the CAC score has been published [43–45].

### Statistical analysis

Participant characteristics were summarized with mean and SD or median and IQR for continuous and categorical variables, respectively. We used generalized estimating equation (GEE) models to estimate associations between lipid profiles at each CARDIA visit from Y0 to Y15 as well as cumulative lipid levels from Y0 to Y15 and GAA at both Y15 and Y20 in the same model by including a time term. Given the secular trend (both chronological and biological changes) of lipid profiles during the CARDIA visits, we standardized our lipid measures using the visit-specific standard deviation to make the effects more comparable across study years. We also performed separate analyses stratified by sex and race. Next, we used multiple logistic regression models to estimate the associations between lipid profiles from Y0 to Y20 and Y25 CAC as well as GAA at Y20 and Y25 CAC. Finally, we conducted causal mediation analyses using the R package ‘mediation’ [46]. We assessed the extent to which GAA mediated the association between lipid profiles and CAC. In the mediation analysis two models were fit, one modeling the effects of lipids on GAA, and a second one jointly modeling the effects of lipids (directly) and GAA (indirectly) on CAC. Using Monte Carlo simulations, a mediation proportion was estimated, indicating how much of the effects of lipids on CAC could be explained by the indirect path in which lipids drive a change in GAA, which then affects CAC. This effect was estimated in a model including confounding variables that affected both the mediating variable (GAA) and the outcome (CAC). All models adjusted for study center, sex, race, education, alcohol consumption, and physical activity. BMI was further adjusted as a sensitivity analysis. All analyses (except for the mediation analysis) were performed using SAS (version 9.4, SAS Institute). Two-sided tests were used throughout, and *P* values less than 0.05 were considered statistically significant.

## Supplementary Information

**Additional file 1.** Trend of lipid profiles by CARDIA study year.

## Data Availability

Data used in preparation of this article were obtained from the CARDIA database (cardia.dopm.uab.edu).

## Abbreviations

ASCVD: Atherosclerotic cardiovascular disease
CAC: Coronary artery calcification
CARDIA: Coronary Artery Risk Development in Young Adults Study
DNAm: DNA methylation
EEAA: Extrinsic epigenetic age acceleration
GAA: GrimAge acceleration
GEE: Generalized estimating equation
GOLDN: Genetics Of Lipid Lowering Drugs and diet Network study
HDL-C: High-density lipoprotein cholesterol
LDL-C: Low-density lipoprotein cholesterol
TG: Triglycerides
WHI: Women's Health Initiative
