# Supplementary figures and images for: Plasma lipid profiles in early adulthood are associated with epigenetic aging in the Coronary Artery Risk Development in Young Adults (CARDIA) Study
Source: Clin Epigenetics. 2022 Jan 31;14:16. doi: 10.1186/s13148-021-01222-2 (PMC8805309; doi:10.1186/s13148-021-01222-2)

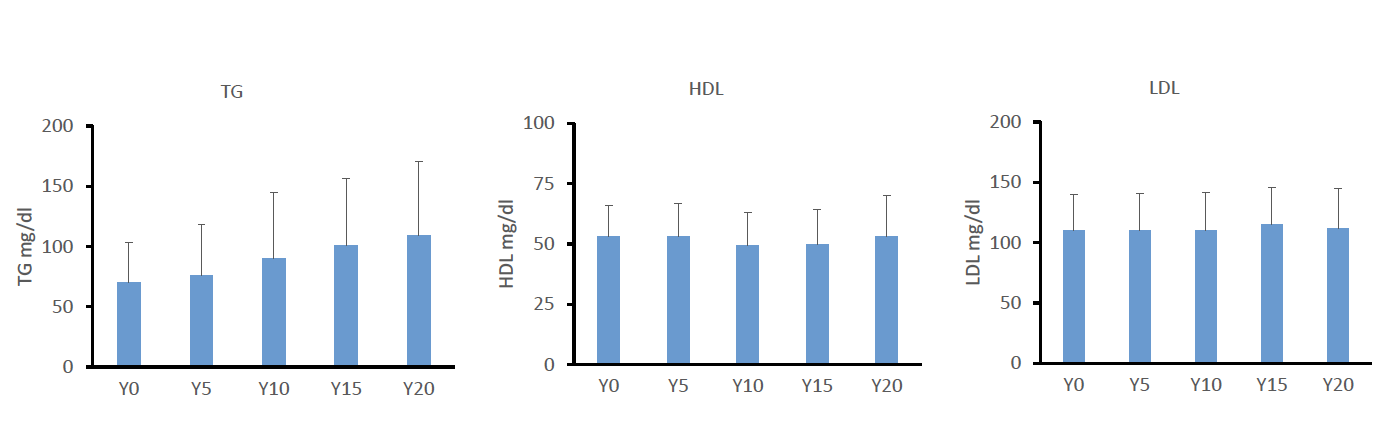
**Supplemental figure 1. Trend of lipid profiles by CARDIA study year(n=1118).**

Supplement: Supplementary file 1 — Additional file 1. Trend of lipid profiles by CARDIA study year. [file 13148_2021_1222_MOESM1_ESM.docx]
